# The Pathological Society

**Published:** 1899-11-11

**Authors:** 


### THE PATHOLOGICAL SOCIETY.

The first of the "laboratory meetings" of the Pathological Society of London was held on Tuesday at the Jenner Institute, and was a great success. A large number of Fellows and visitors attended. The chair was taken by the President (Mr. Watson Cheyne), and many very interesting matters were brought forward for demonstration and discussion. In deference to the very wise ruling of the Council that these meetings shall not be reported, we refrain from saying more than that, while the "show" was of that highly technical character which is so dear to those who spend their days in the study of bacteriology, and their nights at scientific meetings, confounding the outer philistines, and sometimes each other, with their marvellous discoveries, there was much in the discussions which was highly suggestive even to those whose time is mostly filled with the more practical and human side of medical work. What we must not omit to do, however, is to say a word of praise and express our high appreciation of the splendid laboratories in which the meeting was held. Of course, it is all new, and, like many new things, will improve. The theatre, with its smooth walls and floor, and benches, is a trifle resonant, making the speeches not always too easy to hear, while magnifying footsteps, coughs, and other abnormal sounds; then, perhaps, before another lecture is given a "pointer" will be found, and we shall not see the demonstrator waving a window-rod and casting shadows as of a great boat-hook on the screen; and doubtless by then, also, the electric lantern will have learned its lesson; for it must be confessed that on this occasion, like the young lady's curl, when it was good it was very good, but when it was bad it was horrid. Nevertheless it was a capital meeting. Dr. Church proposed a vote of thanks to Lord Lister, the President of the Institute, for allowing the meeting to take place there, and to Dr. Macfadyen for the trouble he had taken in the matter, and in so doing he gave a sketch of the development of pathology and of the steady enlargement of the scope of the society. The motion was seconded by Mr. Bryant, and carried with acclamation, and in replying Lord Lister expressed the hope that this would be by no means the last time that the society would visit the institute.

